# A bubble-free strategy for saline-immersion endoscopic submucosal dissection: optimized electrosurgical settings and continuous low-pressure saline perfusion

**DOI:** 10.1055/a-2780-1100

**Published:** 2026-02-03

**Authors:** Masafumi Kitamura, Yuji Ino, Kosei Hashimoto, Hisashi Fukuda, Mayo Tanabe, Haruhiro Inoue, Hironori Yamamoto

**Affiliations:** 1 12838 Department of Medicine, Division of Gastroenterology, Jichi Medical University, Shimotsuke, Japan; 2 378609 Digestive Disease Center, Showa Medical University Koto Toyosu Hospital, Tokyo, Japan; 3 12838 Department of Endoscopic Research and International Education Funded by FUJIFILM Medical Co., Ltd, Jichi Medical University, Shimotsuke, Japan

Saline-immersion therapeutic endoscopy and the water pressure method have recently gained attention as novel approaches for endoscopic submucosal dissection (ESD 1 2 ). These techniques offer multiple advantages, including stable visualization of the submucosal layer, improved scope maneuverability, and controlled submucosal expansion by buoyancy and hydrostatic pressure. However, during saline-immersion ESD, visibility is often impaired by bubble formation resulting from vaporization and electrolysis during the use of high-frequency devices 3 .

**Fig. 1:**
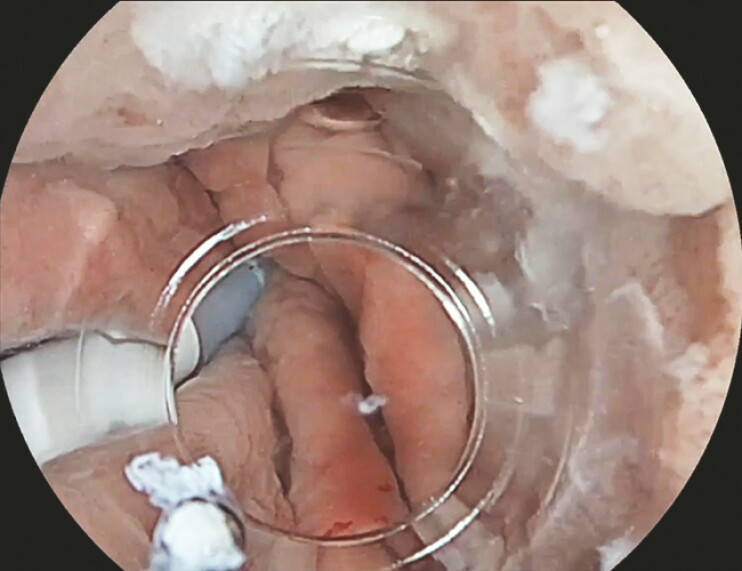
A nasogastric tube is in place for continuous low-pressure saline perfusion (CLPSP).

To overcome these issues, we developed continuous low-pressure saline perfusion (CLPSP; Fig. 1 4 5 ). CLPSP rapidly expels bubbles from the endoscopic field, maintaining a clear view even during active dissection. Nevertheless, when high-voltage modes such as swiftCOAG and forcedCOAG are applied, which are often applied during submucosal dissection to achieve hemostasis, the generation of bubbles remains pronounced because of spark-induced vaporization and electrolysis. Even with continuous saline perfusion, these bubbles can temporarily impair visibility.

**Fig. 2:**
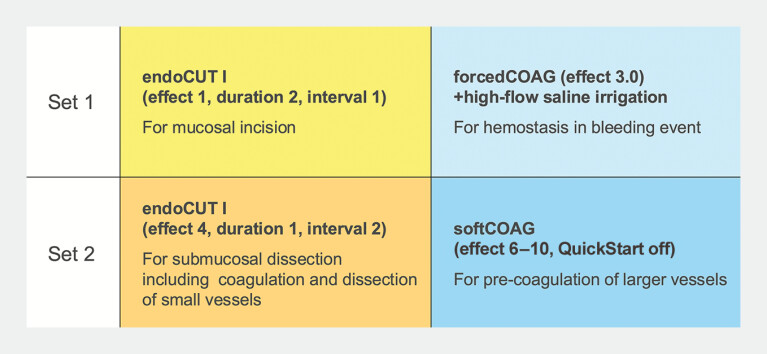
Optimized high-frequency settings used for bubble-free ESD under saline immersion. ESD, endoscopic submucosal dissection.

**Fig. 3:**
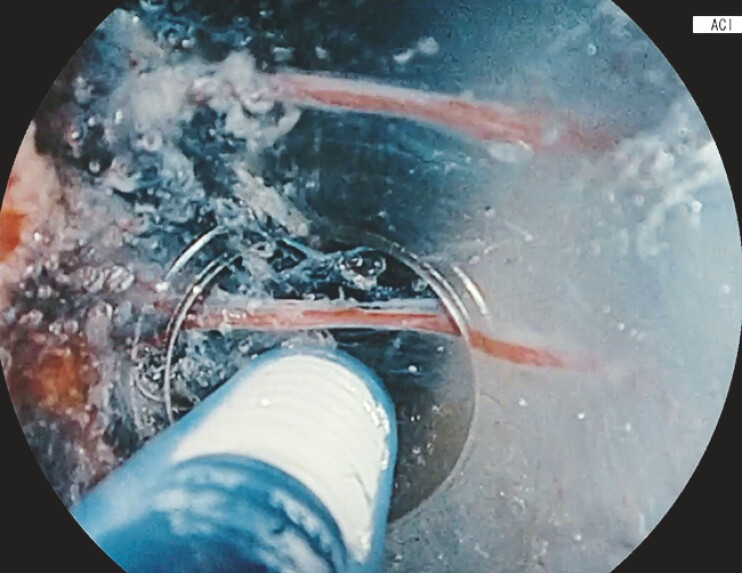
Combination of optimized high-frequency settings with CLPSP enables bubble-free ESD under saline immersion, maintaining a clear view even during active dissection. CLPSP, continuous low-pressure saline perfusion; ESD, endoscopic submucosal dissection.

**Fig. 4:**
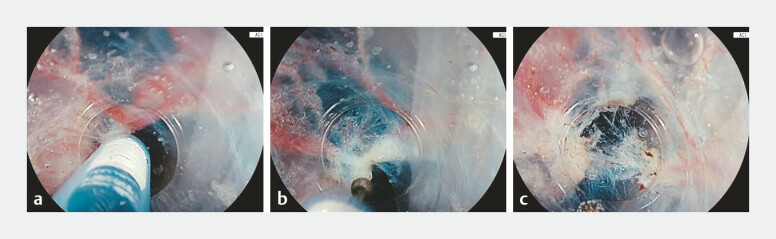
Pre-coagulation of larger vessels using softCOAG under saline immersion. Stable current conduction allows uniform coagulation without bubble formation. **a** Large submucosal vessel. **b** Pre-coagulation using softCOAG (effect 6–10, QuickStart off). **c** Bleeding prevention and safe cutting during dissection.

Based on these findings, we designed an optimized electrosurgical strategy for bubble-free ESD under saline immersion ( Fig. 2 , Fig. 3 and Video 1 ). All procedures were performed using a VIO3 electrosurgical generator (Erbe Elektromedizin, Tübingen, Germany). Submucosal dissection is performed using endoCUT I (effect 4, duration 1, and interval 2), whereas softCOAG (effect 6–10, QuickStart off) is used for pre-coagulation of larger vessels. Under saline immersion, unlike in air, softCOAG ensures stable current conduction and uniform coagulation, even with a needle-type knife ( Fig. 4 ). SoftCOAG delivers a low-voltage continuous current without spark or bubble formation. In the event of bleeding, high-flow saline irrigation maintains visibility, and forcedCOAG achieves hemostasis while suppressing vaporization and spark formation. In addition, CLPSP using automatic water irrigation synchronized with the electrosurgical unit (EIP2; Erbe, Tübingen, Germany) is useful for this technique.

A bubble-free strategy for saline-immersion endoscopic submucosal dissection: optimized electrosurgical settings and continuous low-pressure saline perfusion.Video 1

This combination of optimized high-frequency settings with CLPSP enables bubble-free ESD under saline immersion, preventing inadvertent bleeding and allowing smooth, efficient, and safe dissection.

Endoscopy_UCTN_Code_TTT_1AO_2AG_3AD
